# Risk Analysis of Prion Diseases in Animals

**DOI:** 10.3201/eid1006.030948

**Published:** 2004-06

**Authors:** Brian W.J. Mahy

**Affiliations:** * Centers for Disease Control and Prevention, Atlanta, GA, USA

**Keywords:** prion diseases, animals, scrapie

Although scrapie, a prion disease of sheep, has been recognized since the 18th century, it was the dramatic emergence of bovine spongiform encephalopathy (BSE) in British cattle in the late 1980s that brought the dangers of prion diseases into prominence. The subsequent spread of this disease into humans as new variant Creutzfeldt-Jakob disease (CJD) remains one of the unsolved emerging infectious disease mysteries of the 20th century. This issue (OIE Scientific and Technical Review 22 [2003]) of the Scientific and Technical Review of the Office International des Epizooties comes 11 years after a previous issue about BSE. Much has happened since then; this multi-author volume provides an excellent account of what is known about prion diseases in animals, including BSE, which has now spread from the United Kingdom to 14 other countries, and remains an important risk to human health. As a consequence of BSE emergence, research has been expanded considerably into transmissible spongiform encephalopathies (TSE). This research has resulted in improved diagnostic tests, which have contributed to risk management, even though our understanding of the underlying molecular pathogenesis of TSE remains limited.

The 17 chapters in this book are written by experts from many countries and illustrate the various approaches to risk management in specific regions of the world. In addition to BSE, the book contains chapters about TSE of North America such as scrapie, transmissible mink encephalopathy, and chronic wasting disease of deer and elk; the last disease has recently spread from its endemic Colorado-Wyoming area to six other states in the United States, as well as Alberta and Saskatchewan in Canada.

With the recognition that BSE had spread in the United Kingdom and many other countries through feeding contaminated mammalian meat-and-bone meal to ruminants, a ban on this practice was instigated in the European Union in 1994. This ban has since been adopted by many other regions of the world. In 2000, the European Union strengthened the ban to prohibit feeding processed animal proteins to farmed animals kept, fattened, or bred to produce food. This measure has undoubtedly helped to prevent or reduce numbers of cases of BSE in ruminants but incidentally has led to a new science-based industry for feed analysis. This industry uses sophisticated molecular techniques such as near infrared spectroscopy and microscopy, polymerase chain reaction, and immunoassays to check feed for animal by-products, and to prevent intraspecies recycling (cannibalism), which undoubtedly caused the BSE epidemic that resulted in the death of 186,000 cattle from 1985 to 2002.

I recommend this book as an excellent source of information on all aspects of prion diseases in animals.

